# Temporally and spatially distinct theta oscillations dissociate a language-specific from a domain-general processing mechanism across the age trajectory

**DOI:** 10.1038/s41598-017-11632-z

**Published:** 2017-09-11

**Authors:** Caroline Beese, Lars Meyer, Benedict Vassileiou, Angela D. Friederici

**Affiliations:** Department of Neuropsychology, Max Planck Institute for Human Cognitive and Brain Sciences, Stephanstr. 1a, 04103 Leipzig, Germany

## Abstract

The cognitive functionality of neural oscillations is still highly debated, as different functions have been associated with identical frequency ranges. Theta band oscillations, for instance, were proposed to underlie both language comprehension and domain-general cognitive abilities. Here we show that the ageing brain can provide an answer to the open question whether it is one and the same theta oscillation underlying those functions, thereby resolving a long-standing paradox. While better cognitive functioning is predicted by low theta power in the brain at rest, resting state (RS) theta power declines with age, but sentence comprehension deteriorates in old age. We resolve this paradox showing that sentence comprehension declines due to changes in RS theta power within domain-general brain networks known to support successful sentence comprehension, while low RS theta power within the left-hemispheric dorso-frontal language network predicts intact sentence comprehension. The two RS theta networks were also found to functionally decouple relative to their independent internal coupling. Thus, both temporally and spatially distinct RS theta oscillations dissociate a language-specific from a domain-general processing mechanism.

## Introduction

Language comprehension in general remains remarkably stable across the lifespan; sentence comprehension, however, is known to decline with age^1, 2^. This age-related decline holds particularly for working memory-intensive sentences^3^, which predominantly tax the encoding and retention of verbal information across increased intervals^3–6^.

The ability to encode working memory-intensive sentences, such as structurally complex, ambiguous, or long sentences^7–9^, is a major determinant of language comprehension. That is, language comprehension requires the encoding and retention of verbal information over either increased hierarchical or sequential distances while simultaneously processing intervening verbal material, potentially interfering with already encoded information. Therefore, the encoding success can be affected quantitatively by the number of words (i.e., capacity-based^3^) as well as qualitatively by the memory strength (i.e., decay-, interference- and structure-based^10–12^). On complex sentences, readers with a high memory span perform faster and more accurately than low-span readers^1, 8, 9, 13^. This difference is supported by electrophysiological studies showing that event-related brain potentials (ERPs) associated with sentence processing difficulty^14^ increase for low-span as compared to high-span readers^9^. Likewise, processing differences have been found during the disambiguation of ambiguous sentences, with larger ERP differences for high-span readers than for low-span readers^7^. Such differences are particularly pronounced when ambiguities are extended^7^, possibly suggesting^3^ that low-span readers’ verbal working memory resources are exhausted by the increased duration of an ambiguity^4, 5, 9, 15^.

Given its critical role for sentence comprehension, the decline of verbal working memory capacity with age^16^ could also underlie the decline in sentence comprehension in the elderly^17^. When sentence processing is working memory-intensive, older adults perform worse than young adults. Syntactically complex sentences are processed slower and less accurately by older as compared to young adults^2, 18–22^. Likewise, older adults comprehend ambiguous sentences less accurately than young adults, behaving much like low-span readers across age groups^23^. Finally, increasing sentence duration also decreases older adults’ sentence comprehension accuracy^18, 19, 24^: Poorer repetition accuracy was found for 8-word sentences compared to 5-word sentences in older but not in young adults^24^.

Despite substantial behavioural evidence for the age-related decline in working memory-intensive sentence comprehension, its neural underpinnings as measured by electrophysiological methods remain highly controversial. In healthy young adults, event-related theta power changes have not only been linked to verbal working memory in general^25, 26^ but also specifically to the verbal working memory demands during sentence comprehension^6, 27^. That means, theta power seems to be modulated both by domain-general and domain-specific working memory demands. For example, during general verbal working memory tasks, increased theta power has been found to predict better performance. In comparison, during sentence comprehension, increased theta power has been found for more compared to less complex sentences^28^ showing that the theta rhythm may be modulated by memory-taxing sentences^29^, too. The increase in theta power may be linked to increased synchronisation within the functional network of working memory^29^. In contrast to those event-related studies mentioned afore, in the resting state (RS), decreases in theta power seem to predict better verbal working memory^30–32^. This may imply that, in the brain at rest, less specificity and more variability in frequency-specific neural synchrony may predict optimal functioning at task. This may be due to better readiness for settling into a new state during task-related processing. This is in line with proposals from previous studies^33^. In sum, that means, there is a critical gap in our understanding of the role of spontaneous neural activity, recorded in the brain at rest, particularly for the functional integrity of the language network. Our study is a first attempt to bridge this gap linking changes in RS theta power to working memory-intensive sentence comprehension.

**Figure 1:**
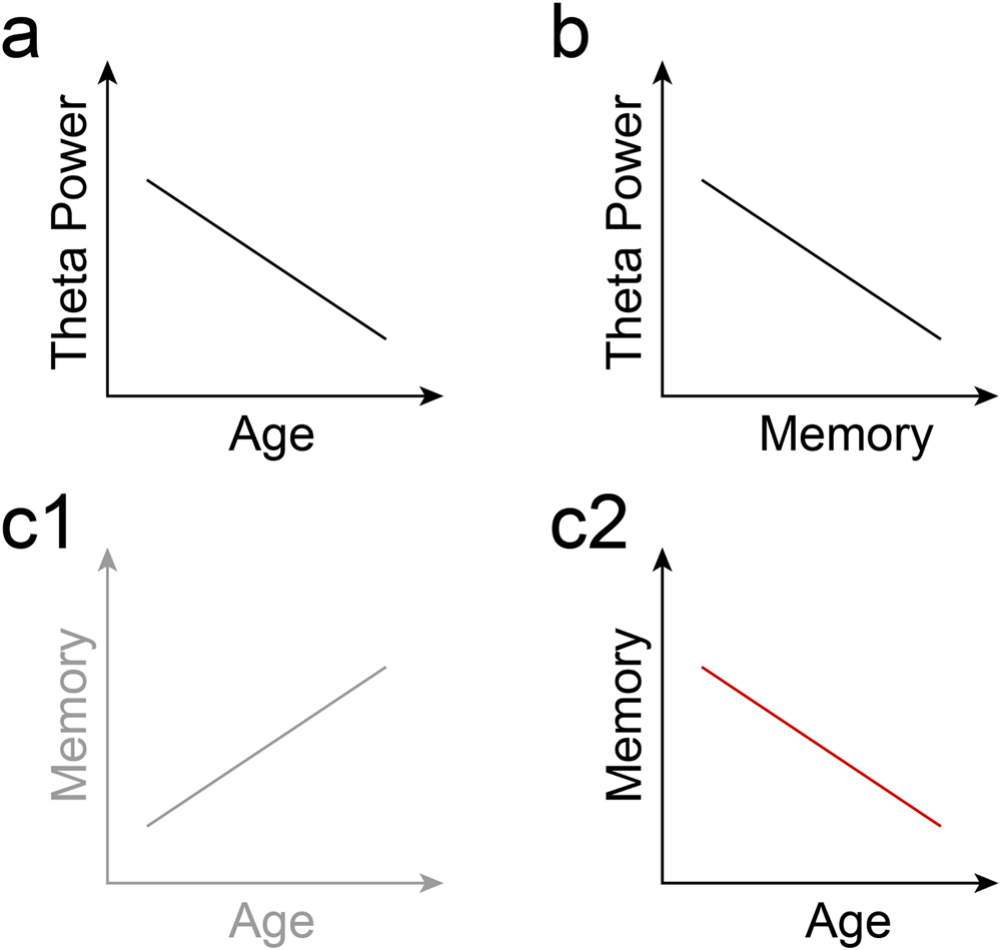
Schematic overview of paradox as a consequence of two coinciding patterns: (**a**) an age-related decrease in resting-state theta power and (**b**) lower resting-state theta power predicting good working memory performance. Taking (**a**) and (**b**) together, one would predict (**c1**), an age-related increase in working memory. However, previous studies found (**c2**) working memory decreases with age.

Given the association between RS theta power and verbal working memory, and given the association between verbal working memory and sentence comprehension across the lifespan, RS theta power could be a plausible lifespan predictor of working memory-intensive sentence comprehension. This hypothesis, however, faces a paradox in the light of three findings (Fig. 1): (a) while RS theta power decreases across the lifespan^34^, (b) and low theta power in the RS indicates good verbal working memory, verbal working memory and sentence comprehension do not improve with age (c1) but instead deteriorate in older adults (c2). Hence, the association between RS theta power, verbal working memory, and sentence comprehension appears to change across the lifespan.

Here we resolve this paradox, extending previous findings^30^: First, we established RS theta as the electrophysiological marker of working memory-intensive sentence comprehension across three age groups (young, middle-aged and older adults). To this end, we correlated RS theta power across the age trajectory with performance on a working memory-intensive sentence comprehension task. Second, we associated age-related decreases in RS theta power to domain-general cognitive abilities supporting sentence comprehension. This approach enabled us to assess the functional integrity of the brain networks underlying working memory-intensive sentence comprehension. Our findings established a crucial role of RS theta power for successful sentence comprehension that is preserved across the lifespan in spite of an age-related cognitive decline.

## Results

**Figure 2:**
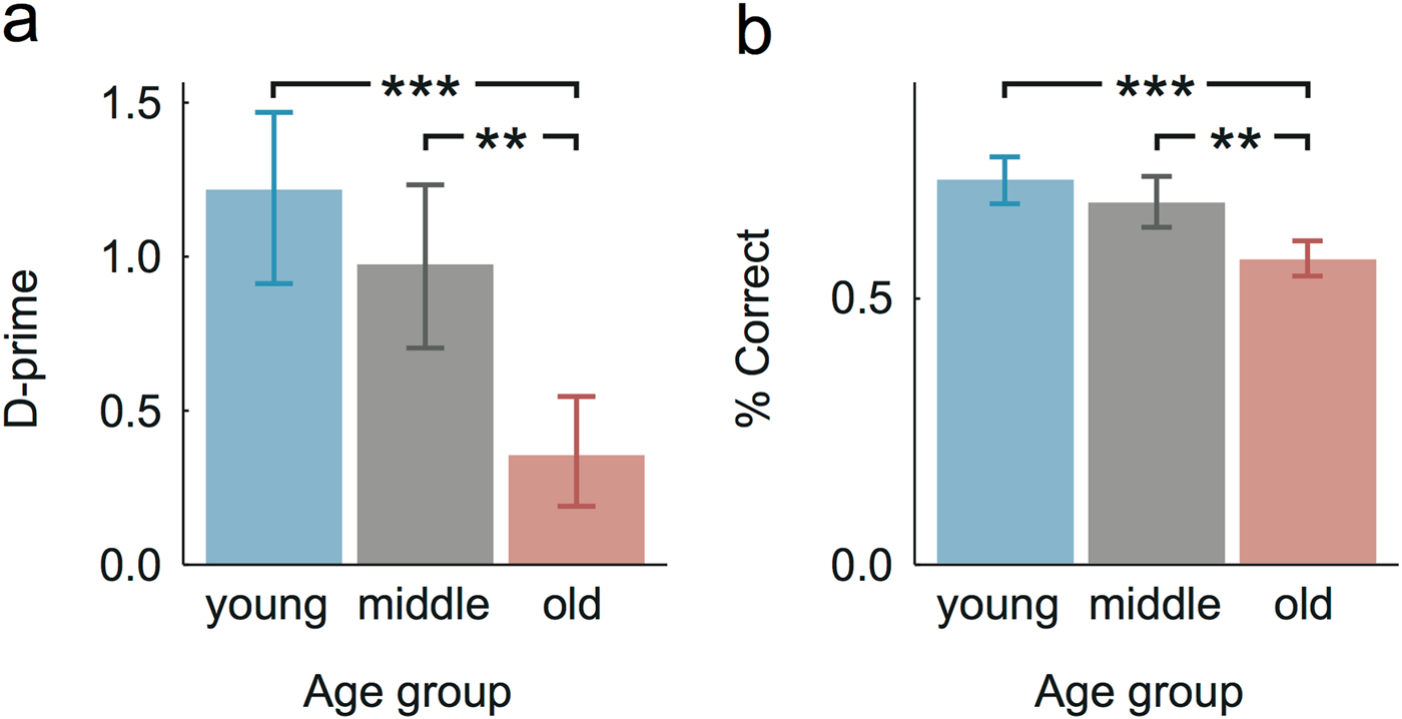
Behavioural results: (**a**) average d-prime scores; (**b**) average percentage of correctly comprehended sentences; both for the three age groups (25, 43 and 65 years of age, error bars indicate the standard error). For both measures, age groups differed in sentence comprehension performance, such that older subjects showed a reduced sentence comprehension performance relative to young and middle-aged adults (*** = p < 0.001; ** = p < 0.01).

The working memory-intensive sentence comprehension task used in this study assessed encoding success through a combination of a retrieval sentence and a comprehension question (see Table 1 and Methods). Behavioural performance was quantified through calculating d-prime scores as well as the percentage of correctly comprehended sentences (Fig. 2).

**Table 1:** Stimulus materials; encoding sentence with examples of both subject and object retrieval sentences as well as comprehension questions and subsequent feedback.

**Phase**		**Example**	
Encoding(5.0–7.8 s, A)		Der Moderator hat den Schriftsteller und die Sängerin angekündigt und die Moderatorin hat den Schauspieler und die Künstlerin angekündigt. *The presenter (m) had announced the writer (m) and the singer (f) and the presenter (f) has announced the actor (m) and the artist (f).*	
		**Subject**	**Object**
Retrieval(3.5 s, V)		Die sie Ankündigende war nervös. *The one (f) announcing her was nervous.*	Die von ihr Angekündigte war nervös. *The one (f) announced by her was nervous.*
	CORR	War die Moderatorin nervös? *Was the presenter (f) nervous?*	War die Künstlerin nervös? *Was the artist (f) nervous?*
Question(< 4 s, V)	GEN-L	War die Sängerin nervös? *Was the singer (f) nervous?*	War die Schauspielerin nervös? *Was the actor (f) nervous?*
	CAT-L	War die Künstlerin nervös? *Was the artist (f) nervous?*	War die Moderatorin nervös? *Was the presenter (f) nervous?*
Feedback (1.0 s, V)			

Note: (f) = female, (m) = male; CORR = correct, GEN-L = gender lure, CAT-L = category lure; A = Auditory, V = visual.

The ANOVA of the behavioural data showed a main effect of age group, F(2,54) = 11.74, p = 5.86 × 10^−5^, η^2^ = 0.30, indicating that d-prime decreases with age (Fig. 2a). Similarly, the ANOVA regressing age group against the percentage of correctly comprehended sentences indicated that sentence comprehension declines with age, F(2,54) = 12.07, p = 4.66 × 10^−5^, η^2^ = 0.31 (Fig. 2b).

In addition to the working memory-intensive sentence comprehension task, a cognitive screening was performed to, on the one side, ensure healthy ageing of our participants and, on the other side, examine domain-general cognitive abilities associated with the working memory-intensive sentence comprehension task. The results showed significant correlations (FDR-corrected) between d-prime and measures of verbal working memory as well as attention (see Methods): the non-word repetition task (r = 0.45, p = 5 × 10^−4^), the digit span forward (r = 0.04, p = 5 × 10^−4^), the digit span backward (r = 0.51, p = 1 × 10^−5^) and the Auditory Flankers Task (r = −0.41, p = 2 × 10^−3^). All of those measures declined with age (non-word repetition: F = 0.45, p = 5 × 10^−4^; digit span forward: F = 0.04, p = 5 × 10^−4^; digit span backward: F = 0.51, p = 1 × 10^−5^; Auditory Flankers: F = −0.41, p = 2 × 10^−3^; Table 2). Given that those domain-general measures are all significantly correlated with the sentence comprehension task, this may suggest a role for the age-related decline in domain-general cognitive abilities in the increased difficulties in working memory-intensive sentence comprehension with age. This proposal is substantiated by our resting state findings.

**Table 2:** Age-related declines in domain-general cognitive abilities; reporting means (standard deviations) as well as the statistical output from the ANOVAs regressing age group against the neuropsychological measures.

Task	Young	Middle	Old	F	p
Digit span task forward	11(2)	11(2)	10(2)	4	0.03
Digit span task backward	10(2)	8(2)	6(2)	17	<0.001
Non-word repetition task	28(3)	28(3)	25(4)	5	0.01
Auditory flankers task	0.01(0.05)	0.05(0.06)	0.10(0.08)	9	<0.001

**Figure 3:**
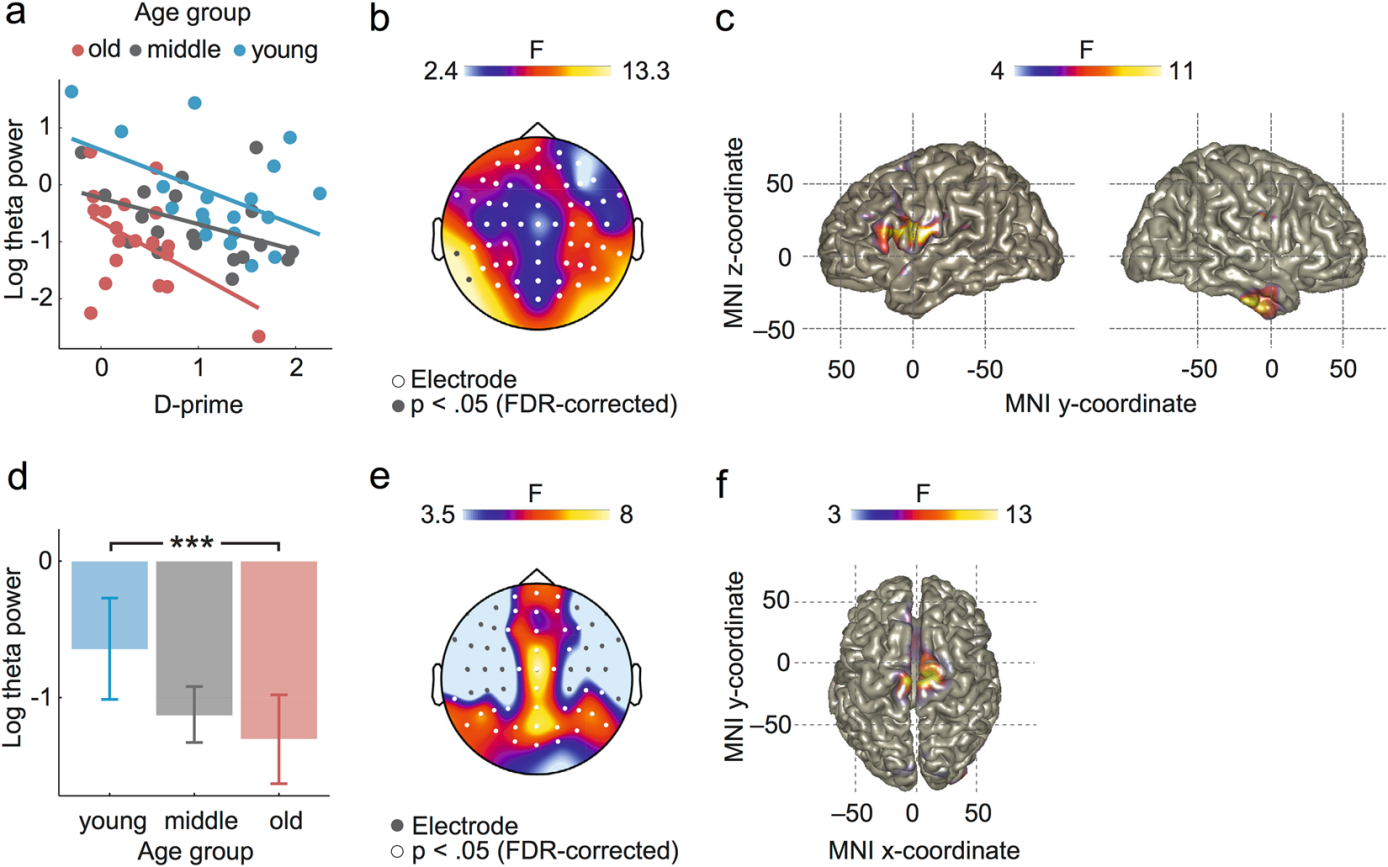
Mechanisms supporting sentence comprehension; (**a–c**) Overview of EEG results supporting a language-specific mechanism (ANCOVA on log RS theta power at each electrode, regressing age group and RS theta power against d-prime) (**a**) scatter plot of log RS theta power at TP9 and P9 predicting sentence comprehension per age group; (**b**) statistical map of the main effect for RS theta predicting sentence comprehension (F is log-scaled) (**c**) source-level results of main effect for RS theta power predicting sentence comprehension (F is log-scaled); (**d-f**): Overview of EEG results supporting a domain-general mechanism (ANOVA on age group predicting log RS theta power): (**d**) average log RS theta power for three age groups (25, 43 and 65 years of age, error bars indicate the standard error). Age groups differed in log RS theta power such that older adults showed reduced log RS theta power relative to young adults (*** = p < 0.001); (**e**) statistical map of the main effect for age group predicting log RS theta power (F is log-scaled); (**f**) source-level results of the main effect for age effect group predicting log RS theta power.

Resting state EEG data from each subject were analysed in the frequency domain using the fast fourier transform (FFT). Statistical analyses were performed hypothesis-driven on the RS data in the theta frequency band. However, to control for effects within neighbouring frequency bands, we also looked at the delta (1–4 Hz), alpha (8–12 Hz), and beta bands (12–25 Hz), none of which showed any additional effects. For source localisation of the theta effect, we used a beamformer in the frequency domain. An analysis of covariance (ANCOVA) on the RS data at each electrode, regressing age group and RS theta power against d-prime, showed main effects of age group at all electrodes (all F(2,51) between 12.92 and 19.91, all p between 4.05 × 10^−7^ and 2.89 × 10^−5^, all η^2^ between 0.33 and 0.44; false discovery rate (FDR) corrected, and main effects of RS theta power (Fig. 3a) at electrodes TP9 (F(1,51) = 12.28, p = 9.62 × 10^−4^, η^2^ = 0.19; FDR corrected) and P9 (F(1,51) = 13.25, p = 6.36 × 10^−4^, η^2^ = 0.20; FDR corrected; Fig. 3b). There were no interaction effects at any electrode (all F(2,51) between 0.80 and 1.68, all p between 0.20 and 0.92) indicating that the relation between theta and d-prime was not affected by any age-related changes. The main effect of RS theta was source localised predominantly to the left-hemispheric dorso-frontal cortex (i.e., at the intersection of the left pre- and postcentral and middle frontal gyrus as well as the pars triangularis) and, less consistently, to the right anterior inferior and middle temporal gyrus (all F(1,51) between 4.05 and 11.34, p between 0.002 and 0.05, uncorrected; Table 3; Fig. 3c).

**Table 3:** Source-level results (p < 0.05, uncorrected) of both the language-specific effect for RS theta power predicting sentence comprehension as well as the domain-general effect for RS theta.

Cluster	Region	F-value (peak)	MNI coordinates (mm)		
			X	Y	Z
Language-specific					
Left	postcentral gyrus	11.34	−66	−5	19
	precentral gyrus	10.80	−56	5	19
	pars triangularis	9.55	−46	25	19
	middle frontal gyrus	6.30	−36	5	49
Right	inferior temporal gyrus	9.91	64	−15	−31
	middle temporal gyrus	7.31	54	5	−31
Domain-general					
Left	medial frontal gyrus	7.29	−6	35	49
	supplementary motor areas	6.92	−6	15	59
	superior frontal gyrus	5.86	−16	15	49
Right	supplementary motor areas	12.65	4	−15	69
	medial frontal gyrus	6.09	4	35	39

Anatomical labels are taken from the Automated-Anatomical-Labeling brain atlas. Cluster peak coordinates are given in Montreal-Neurological-Institute (MNI) space.

The ANOVA at each electrode, regressing age group against RS theta power, showed main effects of age group (Fig. 3d) at 36 centro-frontal and -posterior electrodes (all F(2,54) between 3.76 and 7.92, all p between 9.60 × 10^−3^ and 0.03, all η^2^ between 0.12 and 0.23; Fig. 3e), showing that RS theta power decreases with age. This age-related effect was source-localised to bilateral midline regions (i.e., a region shared by bilateral supplementary motor areas, the left superior frontal gyrus and the right mid-cingulum; all F(2,54) between 3.17 and 12.65, all p between 3.00 × 10^−5^ and 0.05, uncorrected; Table 3, Fig. 3f). To assess whether this effect is related to a more specific cognitive function, a post-hoc ANCOVA was performed at each electrode regressing age group against RS theta power controlling for domain-general cognitive abilities earlier shown to support working memory-intensive sentence comprehension. Indeed, we found that the inclusion of the non-word repetition task in the model dissolved the effect of age group on theta (all F(2,51) between 0.47 and 7.54, FDR-corrected), indicating its relation to age-related theta decreases over midline regions. All other domain-general cognitive measures that were correlated with the experimental task did not impact the relationship between age group and RS theta power.

**Figure 4:**
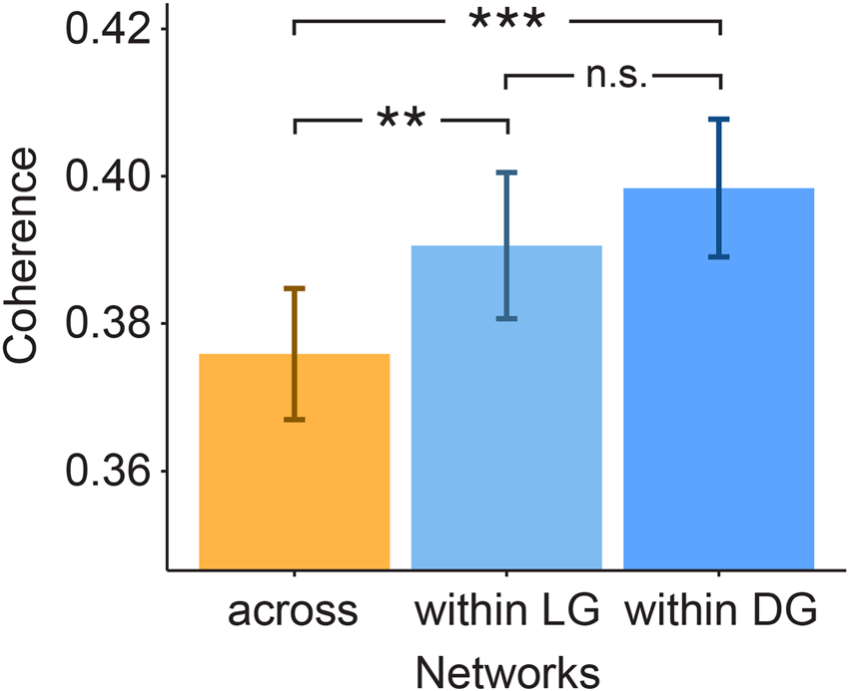
Source-level coherence analysis shows that the coherence of sources within the language-specific network and also within the domain-general network is higher than the coherence of sources across those two networks (*** = p < 0.001, ** = p < 0.01); coherence does not differ significantly across networks (n.s. = not significant; error bars indicate the standard error); LG = language, DG = domain-general.

We reasoned that if the language-specific and the domain-general effect indeed reflect distinct functional networks, subserving distinct cognitive sub-processes of sentence comprehension, the networks should also exhibit a high degree of functional coupling internally, but a significantly lower degree of functional coupling with each other. To follow this post-hoc hypothesis, a source-level coherence analysis on the reconstructed dipole time courses was performed. The results showed that source-level coherence within each of the two networks was higher than coherence across those two networks (internal coherence within the language network: t(56) = −5.91, p = 2.10 × 10^−7^; internal coherence within the domain-general network: t(56) = −4.02, p = 1.75 × 10^−4^). In addition, the coherence of sources within the language network did not significantly differ from the coherence of sources within the domain-general network (t(56) = −1.11, p = 0.27). An additional source separation procedure showed that this result is not confounded by volume conduction but instead the time courses of the two networks cluster around different independent components (z = 3.76, p = 1.68 × 10^−4^). Together, this strongly supports a functional independence of the two resting state networks (Fig. 4).

In sum, we found both a performance-related, language-specific and an age-related, domain-general theta effect with distinct scalp topographies and neural generators. While the scalp topographies already hint at different underlying networks, the source localisation allows for a stronger claim that the scalp-level effects were generated by at least partially non-overlapping brain networks and reflect two distinct mechanisms—one specifically underlying sentence comprehension, the other related to an age-related impairment of domain-general cognitive functions supporting sentence comprehension.

## Discussion

This study addresses a long-standing paradox: Prior research found RS theta power to be negatively correlated with verbal working memory abilities, but also to decrease with age. While this would predict performance in working memory-intensive sentence comprehension to increase with age, this is certainly not the case. Our results resolve this paradox, suggesting that RS theta oscillations dissociate two spatially and temporally distinct networks supporting different mechanisms. On the one side, RS theta power over left-hemispheric dorso-frontal and right anterior temporal regions was found to predict sentence comprehension abilities independently of age. On the other side, we found an age-related decline in RS theta power over central recording sites to be associated with verbal working memory. Moreover, such domain-general cognitive abilities supporting sentence comprehension could be functionally decoupled from the language-specific sentence comprehension abilities. Together this suggests that both older and young adults rely on the same core sentence processing mechanism^35^ located within the dorsal perisylvian language network^36–38^—but that older adults exhibit a performance decline in working memory-intensive sentence comprehension due to changes within the association network subserving domain-general supplementary cognitive functions^22^.

Across the age trajectory, the results revealed an association between working memory-intensive sentence comprehension and RS theta power generated in regions frequently associated with syntactic processing^37–40^: predominantly left-hemispheric dorso-frontal regions and, to a minor degree, the right anterior temporal lobe. This association is independent of age as no interaction was found between RS theta power and age group to explain the behavioural performance. Therefore, while many other cognitive abilities decline with age^16^, the results of the current study suggest that the core sentence comprehension network stays largely intact along the age trajectory. Possibly, the preservation of this performance-related mechanism throughout the lifespan hints at a compensatory role for age-related structural alterations of the language-relevant left-hemispheric dorso-frontal brain regions by functional activation shifts. Previous studies have shown a progressive longitudinal decrease in prefrontal grey and white matter volume across the age trajectory^41^, which coincides with functional activation shifts from posterior to anterior brain regions^42^. To substantiate this proposal, further investigation of the structural neuroanatomy underlying our theta generators is needed.

While the association between dorso-frontal RS theta power and working memory-intensive sentence comprehension can explain the behavioural results within each age group, it does not explain the substantial age-related decline in working memory-intensive sentence comprehension. Instead, we found an indication of spatially and temporally distinct RS theta oscillations that may instead drive this age-related decline in sentence comprehension performance. This effect is most pronounced over the central electrodes, Fz, Cz, Fpz and Pz, with neural generators predominantly in bilateral frontal to central midline regions. We could relate this age-related RS theta power decrease to variations in general verbal working memory performance, more precisely, to variations in the repetition of non-words of increasing syllable length indicating the capacity of the phonological store. Phonological storage, in turn, has been shown to play a role in working memory-demanding sentence comprehension^43^. Therefore, the age-related decline in RS theta power over bilateral frontal to central midline regions associated with working memory differences possibly contributes to the decline in working memory-intensive sentence comprehension. In fact, this is in line with previous findings associating theta over electrode Fz generated by sources along the cingulate as well as the medial frontal cortex with working memory^44–47^. Such frontal midline theta (FMT) was found stronger for high compared to low performers in working memory tasks^48^. More specifically, FMT has been mainly linked to tasks involving order memory, but not item memory^44^. This could explain why the results only showed a link to the non-word repetition task but not to the other working memory tasks included in the cognitive screening (see Methods). The nature of this non-word repetition task involves the memory for the order of phonologically distinctive syllables together constituting individual non-words of increasing length. Hence the order of the syllables is crucial to remember the item. However, generally the link between age-related RS theta power decreases and functional variation in verbal working memory has to be tested more directly by future studies to validate our proposal.

The distinction between the age-related, domain-general and the performance-related, language-specific theta effect can also be observed from their distinct generators. In the mammalian brain, theta-band oscillations can be generated by the hippocampocortical loop—either by the hippocampus itself or by cortical regions within this loop^49^. On the one hand, hippocampal theta, associated with memory formation^50^, may be related to the age-related decline in RS theta power connected to domain-general functioning. On the other hand, cortical regions within the hippocampocortical loop may underlie the language-specific mechanism observed here. This also implies that the location of a theta generator determines its functionality being either language-specific or domain-general. However, this needs further investigation with the help of spatially fine-grained methodologies. Furthermore, it suggests that future studies should use paradigms which manipulate the stimuli in such a way that language-specific processes can be disentangled from domain-general working memory processes. Such paradigms would ensure the dissociation of different underlying oscillatory networks. This way the field of neuronal oscillations could move forward to categorising frequency bands not only by their function but also by their neural generators.

In sum, this study is the first to identify two spatially as well as temporally distinct theta band oscillations subserving distinct functions during sentence processing. The present results show that RS theta power across left-hemispheric dorso-frontal language regions underlies a language-specific mechanism and hence may serve as robust electrophysiological marker of intact sentence comprehension across the lifespan. Our results, moreover, suggest a dissociable domain-general mechanism associated with a decline of supplementary cognitive functions supporting working memory-intensive sentence comprehension—reflected in decreased RS theta power over bilateral midline regions. In sum, we suggest that working memory-intensive sentence comprehension declines with age due to underlying changes in domain-general brain networks, despite the preservation of the core sentence processing network across the lifespan.

## Methods

### Participants

Data from 57 healthy right-handed^51^ participants divided into three equally-sized age groups (young: 9 male; mean age: 25 years; SD: 1 year; middle-aged: 9 male; mean age: 43 years; SD: 2 years; older: 8 male; mean age: 65; SD: 3 years) were analysed. Participants were native speakers of German, had normal or corrected-to-normal vision, and did not suffer from neurological disorders or hearing loss (hearing threshold ≤ 25 dB as assessed by standard audiometry, Oscilla® SM910-B, Aarhus, Denmark). Participants were tested for cognitive impairment (Mini-Mental State Examination 2^52^), assuring that no participant suffered from dementia. Education was matched across age groups (≥14 years of education). All participants gave written informed consent prior to any testing. The study was approved by the ethics committee of the University of Leipzig. The experiment was conducted according to the approved guidelines.

### Neuropsychological Measures

On the first day of testing, all participants underwent a cognitive screening to ensure healthy ageing. The screening included measures of working memory (digit span forward and backward^53^, non-word repetition task^54^, and Counting Span^55^), verbal intelligence (similarities task and vocabulary task), non-verbal intelligence (matrices and block tasks^53^), and auditory attention (Auditory Flankers^56^).

### Stimuli

The experimental items for the working memory-intensive sentence comprehension task were 128 encoding sentences, each consisting of two conjoined clauses containing one subject and two direct objects each. Subjects and objects were all animate in order to avoid associated processing differences^57^. Additionally, subjects and objects differed in grammatical gender within clause, which later served as a retrieval cue. The gender of subjects and objects was counterbalanced across the stimulus set. Subjects and objects were matched for word length (i.e., number of syllables) and word frequency class within and across sentences^58^. This minimised processing differences due to both word length^59^ as well as word frequency^60^. Furthermore, this ensured similar encoding demands for each word, which are affected by both syllable count^61^ and lexical frequency^62^. Subjects’ encoding performance was assessed through a combination of a retrieval sentence and a comprehension question (see Table 1). The retrieval sentence operationalised retrieval via two pronouns, one of the subject’s and one of the object’s gender, thus referring to a unique subject or object within the encoding sentence. In the example retrieval sentence *Die von ihr Angekündigte war nervös* (*The one (she) announced by her was nervous*), the phrase *die von ihr* is pointing to exactly one noun of the encoding sentence. Thereby, *ihr* (*her*) is pointing to the subject, *die Moderatorin* (*the presenter*) who acts upon the female object, *die Künstlerin* (*the artist*), referred to by *die* (*she)*. The subsequent comprehension question assessed retrieval success (and thus, indirectly, encoding success of the encoding sentence) by asking either for the correct item (*Was the artist nervous?*), a gender lure (*Was the actor nervous?*) or a word category lure (*Was the presenter nervous?*). The introduction of lure questions aimed at keeping the amount of correct yes- and no-responses equal. Moreover, the lures necessitated whole sentence processing as category lures emphasised the encoding of both the conjoined clauses whereas gender lures emphasised the encoding of the gender information of all nouns.

Taking into account the number of clauses (i.e., 2), of objects within clauses (i.e., 2) and of subjects per sentence (i.e., 2), 8 variants of each encoding sentence were created in order to counterbalance the gender information of each noun. Those 8 variants were combined with 4 variants of the retrieval sentence varying in gender information of the 2 determiners ensuring that the retrieved items were half male and half female. Hence there were 32 combinations for each of the 128 experimental items which were distributed via Latin Square across 32 lists. This distribution also accounted for the various retrieval types (subject or object retrieval), the retrieval position within the encoding sentence, as well as question types (correct or lure) across lists (within items) and also within lists (across items). With 19 participants in each age group not all 32 variants of each experimental items could be presented. However, this does not pose a limitation as balancing the factors within each list assured equivalent conditions for each participant.

In addition to the experimental items, 64 filler items were included to avoid habituation effects and the buildup of experimental strategies. In comparison to the experimental items, the encoding sentences of the filler items were syntactically more complex (i.e., object-relative clauses and topicalisations). In order to further maximise the difference to the experimental items where gender information was introduced in form of suffixes (e.g. act-or – act-ress), in filler sentences gender information was introduced in form of nouns of biological gender (e.g. uncle – aunt). However, while syntactic differences were maximised, other features like syllable count and word frequency were matched to the experimental items in order to disguise the interests of this study. Furthermore, all content words across experimental and filler stimuli were uniquely used in order to avoid memory consolidation effects, which could have confounded the results. The 64 filler items were added to the 128 experimental items for each of the 32 lists, which were then pseudo-randomised.

### Procedure

The experiment was conducted over two days within a period of no more than 7 days. On the first day, participants underwent audiometer testing and cognitive screening (see Neuropsychological Measures). On the second day, we recorded the electroencephalogram (EEG) at rest and then during the working memory-intensive sentence comprehension task. The sentence comprehension task involved an encoding and a retrieval sentence as well as a comprehension question, and feedback to the subject (see Table 1), in this order. Auditory stimuli were presented via headphones (Sennheiser, HD202). To ensure the same hearing level for all participants, the volume was adjusted to 38 dB above the individual hearing threshold (as determined by the method of limits^63^ on the day of the experiment before setting up the EEG). Visual stimuli were presented in white font (Arial, size 30) on a grey screen (17 inch, Sony Multiscan E220). An experimental trial started with the auditory presentation of an encoding sentence followed by a jittered pause of 1.0–1.5 seconds. A visual retrieval sentence was then presented, querying either a subject or an object from the preceding stimulus sentence (Table 1). A jittered pause of 1.5–2.0 seconds followed. A comprehension question followed, asking for the correct answer (CORR; Table 1) in 50% of trials, for a gender lure (GEN-L; Table 1) in 25% of trials and for a syntactic category lure (CAT-L; Table 1) in 25% of trials. The participants had a response time window of 4 seconds to respond with either “*yes*” or “*no*” by pressing either of two single-button response boxes placed individually under their left and right index finger. Button assignment was counterbalanced across participants. After the response was given, visual feedback was provided in form of a happy or sad emoticon. A trial ended with a jittered inter-trial interval of between 1.5–2.0 seconds.

### Data acquisition

Resting state EEG from each subject was acquired for 5 minutes with the subject’s eyes closed (EC) followed by 5 minutes with the subject’s eyes open (EO), with eye opening prompted by a pure tone of 500 Hz presented at 85 dB. EC and EO were both recorded to enable the calculation of subjects’ individual theta frequency (see Data analysis). Data were acquired within a pass-band from DC to 270 Hz at a sampling rate of 1,000 Hz from 63 electrodes. The setup was referenced against the left mastoid and grounded to the sternum. The vertical and horizontal electro-oculogram (EOG) was acquired with bipolar electrodes below and above the right eye as well as at the outer canthi of both eyes, respectively. The scalp electrodes were placed according to the international 10–20 system in an elastic cap (Electro Cap International, Inc., Eaton, OH, USA) connected to a 72-channel Refa amplifier (TMS International B.V., Odenzaal, The Netherlands). Electrode impedances were kept below 5 kΩ.

### Data analysis

All analyses were performed in MATLAB^®^ (The MathWorks, Inc., Natick, MA, USA). EEG data were analysed using the Fieldtrip toolbox^64^. First, two differently filtered data sets were generated from the raw RS EEG data (EC and EO): First, a band-pass filtered data set for statistical analysis (zero-phase two-pass fourth-order 0.5–45 Hz Butterworth filter); second, a high-pass filtered data set, optimal for independent component analysis (ICA; zero-phase finite-impulse-response one-pass 3624th-order 1-Hz Kaiser filter^65^). Both data sets were re-referenced to the average of all electrodes (excluding the EOG) and segmented into 2-second epochs to facilitate artefact detection. Artefact detection was performed on the high-pass-filtered data set, involving a two-step procedure: First, muscle artefacts were detected using a semi-automatic, distribution-based approach (z > 9) and rejected after visual inspection (EC: mean rejection rate = 8.55%, SD = 7.36%; EO: mean rejection rate = 11.22%, SD = 6.97%), taking into account the waveform morphology. In a second step, the data were down-sampled to 300 Hz to accelerate the subsequent ICA. The first 40 independent components (IC) were calculated and those reflecting heartbeat, eye movements or eye blinks as well as electrode noise were detected based on visual inspection of the components’ waveform morphology, power spectrum, and scalp topography. Artefact ICs were removed from the band-pass filtered data (mean number of rejected ICs = 11.70, SD = 1.99; same for all age groups, F(2,56) = 2.26, p = 0.11), which thereafter was used for all further analyses. After removing A1 and A2 due to leftover noise, the data were re-referenced to the average of all remaining electrodes, and the mean potential within trial was subtracted. The data set was then split into EC and EO. The number of RS trials was pseudo-randomly reduced to the common minimum of 90 trials across all participants—first, trials from the first 30 seconds of the recordings were chosen, and then, trials from the remainder of the data were selected randomly. Using a Hanning taper, frequency analysis via FFT was then performed in steps of 1 Hz from 1–25 Hz on the EC and EO data. Oscillatory power was log-transformed. Because of substantial inter-individual variance in individual band peak frequencies within and across age groups^66^, each subject’s individual alpha peak frequency was detected in order to determine the individual frequency range of the theta band^26, 67^. First, we preselected an extended alpha frequency range (i.e., 7–14 Hz) and electrodes of interest (Pz, P3, P4, P5, P6, P7, P8, P9, P10, POz, PO3, PO4, PO7, PO8, O1, O2, and Oz) separately for the EC and EO data^68^. Second, the EO power spectrum within this range was subtracted from the EC power spectrum. Third, the maximum peak within the difference spectrum was defined as the individual alpha peak frequency (IAF)^69^. IAF was then used as anchor point to constrain the individual theta frequency band into a range from (IAF – 6 Hz) to (IAF – 2 Hz) (adapted from^26, 70^). Subsequent RS analyses were then performed on the EC data^71^.

The effects (see Results) were source-localised using a frequency-domain beamformer (dynamic imaging of coherent sources, DICS^72^). In a first step, a volume conductor was made based on a template Boundary Element Method (BEM) head model^73^. In a second step, this template head model was joined with template sensor positions in accordance to the electrode set up used in this study to create a forward model within a 1-cm-spaced three-dimensional grid that was used for all participants. In a third step, the spatial filter estimated from the cross-spectral density matrix of each subject the power of the sources along every point of the grid, with a signal-to-noise ratio of 3%. Finally, the Neural Activity Index^74^ was computed to remove the center-of-head bias.

For source-level coherence analysis, three-dimensional time courses of source moments were derived from the single-segment preprocessed EEG data by multiplying the single-segment EEG with the individual spatial filter from the DICS beamforming analysis. The source grid was masked for the significant grid points (p < 0.05, uncorrected), and three-dimensional dipole moments were extracted for each volume-conductor grid point within the language-specific and the domain-general effects, respectively. The time course for each grid point then underwent singular value decomposition, arriving at a single time course per grid point^6, 72^. On these time courses, the same FFT frequency analysis that was used for the sensor-level data was performed, now restricted to the individual theta band. Finally, coherence analysis was performed on the complex Fourier-spectra^75^ for all pairs of dipoles within and across the two source-level effects. However, as coherence between areas decays with increasing inter-area distance due to volume conduction^76^, the results may be confounded by larger inter-network and smaller intra-network source distances. For this reason, a supplementary analysis was performed using a blind source separation algorithm within individual. To this end, first, the single-trial source-level time courses from the coherence analysis were downsampled to 100 Hz to speed up later source separation. Second, the time courses were band-pass filtered (finite-impulse-response two-pass 266th-order filter^77^) to match the individual theta band range. Third, an ICA was used to extract 10 independent components, aiming to show that the grid points of the two separate networks would indeed cluster around two different independent components, indicating their functional dissociation. Fourth, the component patterns were masked for all grid points included in the two networks. For each grid point, we then determined the independent component that was maximally represented at the grid points.

### Statistical analysis

Two performance measures were computed: the percentage of correctly comprehended sentences and d-prime scores. D-prime scores were calculated by subtracting the z-transformed false alarm rate (FA) from the z-transformed hit rate (H) (d-prime = z(H) − z(FA)). FA or H of 0 was corrected by 1/N and FA or H of 1 was corrected by (N-1)/N, with N being the number of trials^78^. Analyses of variance (ANOVAs) regressed age group separately against both performance measures. All further analyses were done on the basis of d-prime, as it provides the more sensitive behavioural measure. D-prime was related to the EEG data to assess the relevance of RS theta power for working memory-intensive sentence comprehension. Analyses of covariance (ANCOVAs) were computed for each electrode with age group as factor and RS theta power as covariate of interest, both predicting d-prime^79^. To assess age-related differences in RS theta power, an ANOVA was computed at each electrode with age group as factor predicting theta power. To account for multiple comparisons, the false discovery rate (FDR^80^) procedure was used to correct p-values across electrodes. Additionally, those age-related differences in RS theta power were related to variations in domain-general cognitive functions. For this purpose, in a first step, the experimental measure, d-prime, was correlated with the performance measures of the cognitive screening. In a second step, ANCOVAs were performed at each electrode regressing age group against RS theta power controlling for those measures significantly related to d-prime (after FDR-correction). Measures dissolving the relation between age group and RS theta power indicate their link to age-related RS theta power differences. Finally, in analogy to the sensor-level data, an ANCOVA was performed, with source power predicting behavioural performance depending on the age group. Similarly, an ANOVA was performed with age group as factor predicting the source power. The anatomical labels for the significant clusters (uncorrected) were determined from the MNI atlas^81^. For statistical analysis of source-level coherences, a series of paired-samples t-test was performed, separately comparing the mean coherence of dipole moments within each network (i.e., internal coherence of all dipole pairs within the language network, internal coherence of all dipole pairs within the domain-general network) to the mean coherence of dipole moments across the two networks (i.e., coherence between all pairs that included one dipole from each the language network and the domain-general network). In addition, we also compared the internal coherence of the language network to the internal coherence of the domain-general network. This comparison was substantiated by an additional statistical analysis on source separated component data. We first assessed the difference in the distributions of independent components across networks using within-participant Wilcoxon rank sum tests. The single-subject test statistics from these tests were then submitted to group-level analysis using a one-sample Wilcoxon signed-rank test, hypothesising that the two networks cluster around two different components.
